# Measuring the equity of inpatient utilization in Chinese rural areas

**DOI:** 10.1186/1472-6963-11-201

**Published:** 2011-08-21

**Authors:** Zhongliang Zhou, Jianmin Gao, Ashley Fox, Keqin Rao, Ke Xu, Ling Xu, Yaoguang Zhang

**Affiliations:** 1 School of Public Policy and Administration, Xi'an Jiaotong University, Xi'an, China; 2 Division of Health Policy and Administration, Yale School of Public Health, Yale University, New Haven, CT, USA; 3 Centre of Health Statistics and Information, Ministry of Health, Beijing, China; 4 World Health Organization, Geneva, Switzerland

## Abstract

**Background:**

As an important outcome of the health system, equity in health service utilization has attracted an increasing amount of attention in the literature on health reform in China in recent years. The poor, who frequently require more services, are often the least able to pay, while the wealthy utilize disproportionately more services although they have less need. Whereas equity in health service utilization between richer and poorer populations has been studied in urban areas, the equity in health service utilization in rural areas has received little attention. With improving levels of economic development, the introduction of health insurance and increasing costs of health services, health service utilization patterns have changed dramatically in rural areas in recent years. However, previous studies have shown neither the extent of utilization inequity, nor which factors are associated with utilization inequity in rural China.

**Methods:**

This paper uses previously unavailable country-wide data and focuses on income-related inequity of inpatient utilization and its determinants in Chinese rural areas. The data for this study come from the Chinese National Health Services Surveys (NHSS) conducted in 2003 and 2008. To measure the level of inequity in inpatient utilization over time, the concentration index, decomposition of the concentration index, and decomposition of change in the concentration index are employed.

**Results:**

This study finds that even with the same need for inpatient services, richer individuals utilize more inpatient services than poorer individuals. Income is the principal determinant of this pro-rich inpatient utilization inequity- wealthier individuals are able to pay for more services and therefore use more services regardless of need. However, rising income and increased health insurance coverage have reduced the inequity in inpatient utilization in spite of increasing inpatient prices.

**Conclusions:**

There remains a strong pro-rich inequity of inpatient utilization in rural China. However, a narrowing income gap between the rich and poor and greater access to health insurance has effectively reduced income inequality, equalizing access to care. This suggests that the most effective way to reduce the inequity is to narrow the gap of income between the rich and poor while adopting social risk protection.

## Background

As an important outcome of health system, equity in health service utilization has attracted an increasing amount of attention in China in recent years [1-3]. Researchers frequently distinguish between income-related inequalities and income-related inequities. Income-related inequality in health utilization refers to the disparities in utilization of health services between different income groups. For an inequality to be interpretable as an inequity, differential need must be taken to into account [4]. A common interpretation of equity in health utilization is that health care ought to be allocated on the basis of health need, rather than on the basis of demographic characteristics such as, income, race, or area of residence [5]. Health services are equitably distributed and utilized when people who have greater need use health services proportionally more than those with less need. The conceptual literature on equity in health utilization distinguishes between horizontal and vertical equity. Horizontal equity is interpreted to mean that persons in equal need of care ought, on average, to be treated the same, irrespective of their socioeconomic status [6]. Vertical equity is the unequal, but equitable, treatment of individuals in unequal levels of need [7]. Because analysis of vertical inequity is problematic the economic literature, much of the empirical work in this area has focused on horizontal equity. If an equitable system is one that would allocate more resources towards the neediest segments of society, then poor, rural residents should receive more services in proportion to their need.

As a rapidly industrializing middle income country, the widening income-related inequity in access to health services among residents has been a growing concern of the Chinese government. Whereas the equity in health service utilization in urban settings has received a great deal of attention, far less attention has been paid to health services equity in rural areas. Yet, in spite of large amounts of internal migration, China remains a largely rural country. With more than 50% of people living in rural areas [8], the equity in health service utilization of rural residents is of increasing concern to China's national equity in health service utilization. Further, in spite of economic growth over the last thirty years, large income gaps between urban and rural residents remain. The per capita income for rural residents is less than one third of urban residents [8]. Whereas extreme poverty is on the decline in urban areas, continued high levels of entrenched poverty make rural residents more vulnerable to an array of diseases. With wide gaps between rich and poor in rural areas, increasing health service utilization may be more urgent for poor rural residents than for poor urban residents as a means of improving health status and reducing health inequities.

Furthermore, among rural residents, the inequality of inpatient utilization is even higher than the inequality of outpatient utilization. Poorer individuals use disproportionately fewer in-patient services, which tend to be more costly, compared with wealthier individuals. For instance, when all residents are divided into five equal groups after being ranked by income, the annual hospitalization rates for the richest rural people is 14.3% more than that for the poorest people, while the same number of outpatient rates for two weeks is only 1.4% [9] though little is understood about why this is the case. Previous studies have found that low income is the main reason that prevents the poor from having access to hospitalization [10,11]. Thus, compared with outpatient utilization, the inequality of inpatient utilization requires greater attention. For these reasons, the Chinese government implemented the New Cooperative Medical Scheme (NCMS) for rural residents in 2004. NCMS is a community-based rural health insurance scheme that incorporates two important policy features: voluntary enrollment and coverage of catastrophic illnesses. With the objectives of protecting households from medical impoverishment and improving the equity of inpatient utilization, the target of NCMS is to protect against "catastrophic" inpatient expenses, under the rationale that most households generally are able to afford expenses of minor illness. Thus, although NCMS should have little impact on equalizing routine outpatient visits, the plan should have a substantial equalizing effect on inpatient visits.

Previous studies have indicated that income level, the growth of medical price and medical expenditure payment methods are three main determinants that influence Chinese health service utilization [12,13]. Rising income levels, decreasing medical prices and the implementation of health insurance are factors that should increase health service utilization. Over the past decade, these factors have changed a great deal in rural China. From 2000 to 2009, China experienced rapid economic growth, leading to improved income for a large number of citizens. With an average annual growth rate of rural residents' income of 9.6%, the benefits of economic growth have not been limited to urban areas [8,14]. However, whereas improved income should increase rural residents' ability to pay, the rising cost of care in rural areas may offset the benefits of socioeconomic development. For instance, the inpatient cost of health services for Chinese rural residents has been rising at an alarming rate with an average growth rate of 7.0% from 2000 to 2009 [15,16]. Increasing coverage of health insurance should off-set these rising health care costs to some degree. Yet, in 2000 less than 20% of rural residents were covered by a health insurance scheme [17]. In order to make up for this rising cost of care, and to pool risk more equitably, in 2004, the national government implemented the New Cooperative Medical Scheme, which has covered 94% of rural residents as of 2009 [16]. While insurance should theoretically equalize access to health services across income groups by removing ability to pay as a barrier to care, equality of access may not always translate directly into equality of utilization. Other barriers to access such as distance to health clinics, lack of transportation or preferences for traditional medicine may also affect income-related inequalities in health service utilization. Given these changes in the determinants of health service utilization in rural areas over the last decade, studies are needed on how these developments have affected the inequality of inpatient utilization in rural China and whether the remaining inequality is inequitable. If inpatient utilization differs between different income groups, but these differences are proportional to need, then these differences are not inequitable. If, on the other hand, health service utilization is lower among those who require proportionally more or the same amount of services, this inequality can indeed be considered inequitable. The concentration index is a means of measuring the level of inequity of health services that allows various components of inequality to be decomposed.

Because country-wide data has until recently been unavailable, previous studies have shown neither the full extent of inpatient utilization inequity nor by how much various determinants contribute to inequity in rural areas [18,19]. Furthermore, with only aggregated regional data available, most studies have focused on income-related inequality- whether there are income differences in utilization rates- rather than income-related inequity based on need [20,21]. The study of equity in inpatient utilization for Chinese rural residents using country-wide data goes beyond previous studies to examine the inequity of utilization rates across all rural areas over time.

The purpose of this study is to analyze the present degree of horizontal equity in Chinese rural residents' inpatient utilization, the contributions of the primary determinants of utilization to level of equity and the contributions of determinants to the increasing or decreasing equity from 2003 to 2008. These findings can be used to make recommendations on how to improve the equity of health utilization for rural residents in rapidly developing countries.

## Methods

### Data

The data utilized in this study are from China's National Health Services Surveys completed in 2003 and 2008. These surveys, organized and directed by the Centre for Health Statistics and Information of the Chinese Ministry of Health, were conducted in China for both urban and rural areas in 1993, 1998, 2003 and 2008. This paper uses only the data generated from household questionnaire interviews carried out among the rural residents. The surveys include variables on demographics, income variables, health status, medical service utilization and medical expenses.

A four-stage stratified random sampling procedure and method was used in the household survey in order to achieve maximum representation of the demographic and socio-economic characteristics of the whole population. In the first stage, all the counties in the rural area were grouped into 5 groups according to 10 socio-economic indicators and counties were selected randomly in each group: 67 in 2003 and 64 in 2008. In the second stage, five townships in each county were randomly chosen. In the third stage, two villages in each township were selected. Finally, 60 households were identified in each village. The total number of rural households sampled in 2003 and 2008 were 143,991 and 129,301 respectively. Only the people who were 15 years old and older were selected in this study and the sample sizes are 112,116 in 2003 and 103,754 in 2008.

### Methods to measure health utilization inequality and inequity

The concentration index (CI) is employed in this paper to measure health utilization inequality. The concentration index, which quantifies the degree of income-related inequality in a health variable [22,23], is becoming a standard tool for the measurement of income-related health inequality [24]. The concentration index is zero if there is no income-related inequality of health utilization. If the concentration index takes on a positive (negative) value, there is a pro-rich (pro-poor) inequality in inpatient utilization. The general formula for the concentration index defines it in terms of the covariance between the health variable and the fractional rank in the income distribution [25].

(1) $$C=\frac{2}{\mu }\text{cov}\;\left(y,r\right)$$

Where *C *is concentration index; *y *is inpatient utilization index; *μ *is the mean of inpatient utilization index and *r *is the fractional rank in the income distribution.

The method of decomposition of the concentration index is used to analyze the contributions of various determinants of utilization to the inequality in inpatient utilization and to calculate horizontal inequity. Decomposition of the concentration index, proposed by Wagstaff [26], is a straightforward way to decompose the measured degree of inequality into the contributions of various explanatory factors. According to this method, the concentration index of inpatient utilization can be decomposed into contributions of determinants to income-related inequality using the method of decomposition of concentration index [26]. In keeping with previous studies, the inequality of inpatient utilization is decomposed into four components in this paper [4]: (1) the contribution of individual income; (2) the contribution of the need variables; (3) the contribution of other explanatory variables (i.e., inpatient price); (4) the contribution of the residual term which captures the degree to which the residual is correlated with income rank. In order to decompose the inequality of inpatient utilization, a regression model should be given by

(2) $$y_{i}=\alpha^{}+\beta_{m}x_{}^{m}+\sum_{n}\beta_{n}x_{i}^{n}+\sum_{p}\beta_{p}x_{i}^{p}+\varepsilon_{i}$$

Where *y_i _*is inpatient utilization; *x^m ^*is income; *x^n ^*are need variables; *x^p ^*are other variables; *β_m_*, *β_n _*and *β_p _*are coefficients; *ε_i _*is the implied error term, which includes approximation errors. Then the concentration index for *y *can be written as

(3) $$\begin{array}{c} Ĉ=\left(\beta_{m}{\bar{x}}_{m}∕ȳ\right){Ĉ}_{m}+\sum_{n}\left(\beta_{n}{\bar{x}}_{n}∕ȳ\right){Ĉ}_{n}\\ +\sum_{p}\left(\beta_{p}{\bar{x}}_{p}∕ȳ\right){Ĉ}_{p}+GC_{\varepsilon }∕ȳ \end{array}$$

Where  is the concentration index of inpatient utilization, and ,  and  are the concentration indexes of *x^m^*, *x^n ^*and *x^p^*. The first term of the right side of equation 3 denotes the contribution of income to inequality, the second denotes the contributions of need variables, the third denotes the contributions of other variables and the last term is the generalized concentration index of *ε_i_*. The horizontal inequity (HI) of inpatient utilization can be computed by subtracting the contribution of need variables from the concentration index of inpatient utilization [26].

In order to explain changes in income-related inequality in health service utilization over time, the method of decomposition of change in concentration index, as proposed by Wagstaff [26], is used to decompose the change of concentration index in inpatient utilization from 2003 to 2008. The change of concentration index was further decomposed to assess the contributions of different determinants of health service utilization (see equation 4).

(4) $$\begin{array}{c} dC=-\frac{C}{ȳ}d\alpha +\sum_{k}\frac{{\bar{x}}_{k}}{ȳ}\left(C_{k}-C\right)d\beta_{k}+\sum_{k}\frac{\beta_{k}}{ȳ}\left(C_{k}-C\right)d{\bar{x}}_{k}\\ +\sum_{k}\frac{\beta_{k}{\bar{x}}_{k}}{ȳ}dC_{k}+d\frac{GC_{\varepsilon }}{ȳ} \end{array}$$

Note that the effect on *C *of a change in *β_k_*, or in , depends on whether *x_k _*is more unequally or less unequally distributed than *y*. This reflects two separate channels of influence--the direct effect of the change in *β_k _*(or ) on *C *and the indirect effect operating through .

### Regression models

Two non-linear regression models are employed to decompose the concentration index and to decompose the change of concentration index. Probit regression models are used to analyze the influences of determinants on the probability of an inpatient visit, generalized negative binominal regression models are used to analyze the influences of various determinants of utilization on number of inpatient visits and zero-truncated negative binomial regression models are used to analyze the influences of determinants on per-visit hospitalization days. The linear approximation to the non-linear model is made to estimate the marginal effects evaluated at the means in the process of decomposing concentration index [5].

### Variables

#### (a)Inpatient utilization

In keeping with previous studies on inpatient utilization in China, inpatient utilization is measured in three separate ways: 1. The probability of making an annual inpatient visit; 2. The number of annual inpatient visits; and 3. Per-visit hospitalization days. The probability of an annual inpatient visit refers to the probability of initial use of an inpatient visit in the previous year. Inpatient visits refers to the total number of visits per person per year (including the initial use of inpatient visits and subsequent visits). Per-visit hospitalization days capture the number of hospital days for each episode of hospitalization.

#### (b)Independent variables

In order to be consistent with the method of decomposing the concentration index, independent variables in the regression model are classified into three groups: income, need variables and other variables. Income is measured by self-reported consumption expenditure. Consumption expenditure is used rather than self-reported income because income is more likely to be misreported and consumption expenditure is a better proxy for resources available [27,28]. The contribution of income is defined as the product of the income elasticity in inpatient visits and the concentration index of income. Need is an elusive concept that has been given a variety of interpretations in relation to the definition of equity in health care delivery [29,30]. Here need variables include sex, age and health status. Health status is self-reported by residents, which includes illness in the last two weeks, chronic disease, sick days in last two weeks, days of staying in bed in last two weeks, days off work and study in last two weeks, and a health status index. In 2003, the residents' health status index was divided into five categories: excellent health, good health, average health, poor health, very poor health, while the health status index was measured by scores (ranged from 0 to 100) in 2008.

Other variables include marital status, educational level, occupations, regions, health insurance schemes, time to go to the nearest medical institution, outpatient price and inpatient price. In addition to these standard control variables, we introduce more detailed variables regarding health insurance schemes, outpatient price and inpatient price. In rural China, Cooperative Medical Scheme (CMS) and New Cooperative Medical Scheme (NCMS) (both community-based health insurance schemes) were the main health care insurances for rural people in the year of 2003 and 2008, respectively. In addition, in this paper, outpatient price and inpatient price are measured by the medians of per-visit outpatient expenses and per-visit inpatient expenses at the county level in rural areas.

## Results

### Descriptive analysis

Table 1 shows that, from 2003 to 2008, the probability of an inpatient visit and the number of inpatient visits of Chinese rural residents increased greatly, with growth rates at 82.8% and 92.3% respectively. However, the per-visit hospitalization days decreased slightly, from 10.55 to 10.37. Yet, from 2003 to 2008, rural residents' self-reported health status declined. For example, the probability of being ill in the last two weeks and the probability of having a chronic disease increased by 23.5% and 31.1%. The coverage of health insurance for rural residents also underwent a substantial increase. In 2003, less than 13% residents were covered by health insurance, from which only 9.7% residents were covered by CMS; by 2008, more than 90% residents were covered by NCMS. Compared with 2003, the average outpatient price and inpatient price increased by 84.3% and 34.2% in 2008, respectively. After deflating the per capita consumption expenditure in the year of 2008 to 2003 by using consumer price index, the per capita consumption expenditure in 2003 and 2008 were 2690 Yuan and 4471 Yuan respectively and the growth rate was 66.21%.

**Table 1:** Description of variables in the year of 2003 and 2008 (Percentage/means)

Variable	Description	2003	2008
Inpatient utilization			
Probability of inpatient visit	The probability of seeking inpatient care.	3.37	6.16
Hospitalization rate	Hospitalization rates in the year of 2003 or 2008.	3.89	7.48
Per-visit hospitalization days	Mean per-visit hospitalization days.	10.55	10.37
Independent variables			
Income (RMB)	Mean of per capita consumption expenditure. Natural log of income is introduced in regression models.	2690.32	4471.15
Female*	1 if female, 0 otherwise. Omitted group.	49.61	50.28
Male	1 if male, 0 otherwise.	50.39	49.72
Age 15-34*	1 if age between 15 and 34, 0 otherwise. Omitted group.	37.52	30.96
Age 35-44	1 if age between 35 and 44, 0 otherwise.	21.46	22.69
Age 45-54	1 if age between 45 and 54, 0 otherwise.	19.74	19.19
Age 55-64	1 if age between 55 and 64, 0 otherwise.	10.86	14.95
Age 65+	1 if age between 65 and above, 0 otherwise.	10.42	12.21
Not ill in last two weeks*	1 if not ill in the last two weeks, 0 otherwise. Omitted group.	85.12	21.62
Illness in last two weeks	1 if ill in last two weeks, 0 otherwise.	14.88	18.38
Not Chronic disease*	1 if not chronic disease, 0otherwise. Omitted group		
Chronic disease	1 if chronic disease, 0 otherwise.	13.19	17.29
Sick days (day)	Sick days in last two weeks	7.89	8.52
Bed days (day)	Days of staying in bed because of illness in last two weeks.	1.30	1.16
Off-work days (day)	Days off work and study in last two weeks because of illness.	1.80	1.43
Excellent health*	1 if very good health, 0 otherwise. Omitted group.	38.49	--
Good health	1 if good health, 0 otherwise.	36.34	--
Average health	1 if health, 0 otherwise.	19.99	--
Poor health	1 if bad health, 0 otherwise.	4.54	--
Very poor health	1 if very bed health, 0 otherwise.	0.65	--
Health status index	Mean scores of health status.	--	80.66
Unmarried*	1 if unmarried, 0 otherwise. Omitted group.	18.37	16.52
Married	1 if married, 0 otherwise.	74.8	75.27
Divorced	1 if divorced or separated, 0 otherwise.	0.73	1.21
widowed	1 if widowed, 0 otherwise.	6.10	7.00
Illiterate*	1 if illiterate, 0 otherwise. Omitted group.	22.84	19.04
Elementary	1 if graduating from elementary school, 0 otherwise.	31.17	31.46
Primary	1 if graduating from middle school, 0 otherwise.	36.06	37.84
High	1 if graduating from high school, 0 otherwise	8.85	10.22
University	1 if graduating from university, 0 otherwise	1.09	1.45
Unemployment*	1 if unemployment, 0 otherwise. Omitted group.	3.95	12.92
farmer	1 if farmer, 0 otherwise.	72.28	64.39
student	1 if student, 0 otherwise.	6.31	7.17
Other occupations	1 if other occupations, 0 otherwise.	17.46	15.52
Eastern region*	1 if eastern region, 0 otherwise. Omitted group.	31.18	30.96
Central region	1 if central region, 0 otherwise.	26.84	27.42
Western region	1 if western region, 0 otherwise.	41.98	41.62
No medical insurance*	1 if no medical insurance, 0 otherwise. Omitted group.	87.49	6.51
CMS/NCMS	1 if CMS or NCMS, 0 otherwise.	9.67	90.24
Other medical insurance	1 if other medical insurance, 0 otherwise.	2.84	3.25
Time (minutes)	Mean time of going to the nearest medical institution. Natural log of income is introduced in regression models.	14.26	13.75
Price of outpatient (RMB)	Median price of outpatient service. Natural log of outpatient price is introduced in regression models.	39.08	72.03
Price of inpatient (RMB)	Median price of inpatient service. Natural log of inpatient price is introduced in regression models.	1472.71	1976.60

Note: * Reference groups. Per capita consumption expenditure in the year 2008 is deflated to the year of 2003 by using consumer price index.

### Income-related inequality and inequity

The concentration indexes of the probability of an inpatient visit, number of inpatient visits and per-visit hospitalization days in 2003 and 2008 were positive and all of them were statistically significant at 5%, which demonstrates that the rich are more likely to utilize inpatient services than the poor in Chinese rural areas. However, as the need of inpatient service has not been taken into account, inequality is not equivalent to inequity. The horizontal inequity indexes in health utilization were calculated by using the method of decomposition of the concentration index. As shown in table 2, all of the horizontal inequity indexes of probability of an inpatient visit, number of inpatient visits and per-visit hospitalization days in 2003 and 2008 were positive, which indicates that inpatient utilization inequities exist for rural residents in China, and that the rich utilize inpatient services more than the poor when they have the same health status (pro-rich inequity). Compared to the inequity of the probability of an inpatient visit, the inequity of number of inpatient visits was higher and both of them were higher than the inequity of per-visit hospitalization days. From 2003 to 2008, the horizontal inequity index of the probability of an inpatient visit and the number of inpatient visits decreased 48.37 and 43.59% respectively, while this index of per-visit hospitalization days increased 29.96%.

**Table 2:** Concentration index and horizontal inequity index in inpatient utilization

	Probability of inpatient visit		Inpatient visits		Per-visit hospitalization days	
			
	CI	HI	CI	HI	CI	HI
2003	0.2236*	0.2386*	0.2360*	0.2487*	0.0996*	0.0841*
2008	0.1145*	0.1232*	0.1310*	0.1403*	0.1160*	0.1093*
Change	-0.1091	-0.1154	-0.1050	-0.1084	0.0164	0.0252

Note: *significant at 5%.

### Decomposition of inequality

**Figure 1:**
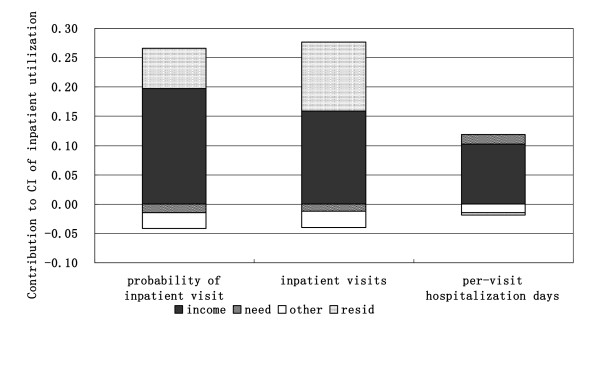
**Decomposition of inequality in inpatient utilization in the year of 2003**.

**Figure 2:**
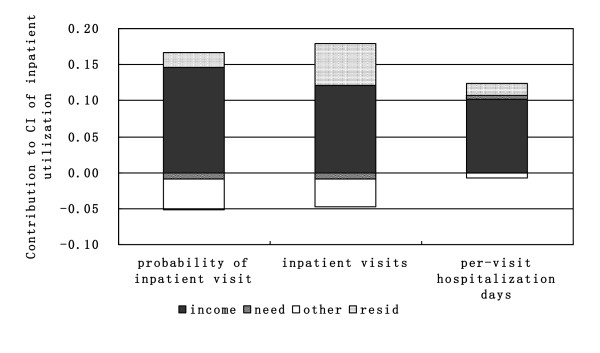
**Decomposition of inequality in inpatient utilization in the year of 2008**.

After decomposing the concentration indexes of inpatient utilization, the income-related inequalities were decomposed into the contributions of different variables (as show in table 3 and table 4). The absolute value of contribution signifies the extant to which inequality can be attributed to this variable. The positive value of contribution means the variable contributes to pro-rich inequality, that is, the richer individuals use more inpatient service than the poor, and vice versa. As the variables were divided into four groups (income, need variables, other variables and residual term), the contribution of each variable-group was generated by adding up contributions of variables within each group (as show in Figure 1 and 2). The sum of the bars would be zero if utilization were equal across income and the need bar would be the only bar to appear if there were perfect equity.

**Table 3:** Decomposition of inequality in the inpatient utilization in 2003

	Prob of inpatient visit		Inpatient visits		Per-visit hospitalization days	
			
	Marginal effects	Contributions	Marginal effects	Contributions	Marginal effects	Contributions
Income	0.0178*	0.1972	0.0165*	0.1586	2.7567*	0.1022
Male	-0.0061*	0.0006	-0.0041*	0.0003	2.4735*	0.0033
Age 35-44	-0.0195*	-0.0172	-0.0180*	-0.0138	2.8649*	0.0054
Age 45-54	-0.0173*	0.0025	-0.0156*	0.0020	3.9752*	0.0019
Age 55-64	-0.0140*	0.0061	-0.0126*	0.0048	2.6040*	-0.0009
Age 65+	-0.0156*	0.0071	-0.0134*	0.0053	1.9663*	0.0000
Illness in last two weeks	0.0000	0.0000	0.0004	0.0000	-1.8330*	-0.0006
Chronic disease	0.0378*	0.0025	0.0389*	0.0022	1.2519*	0.0015
Sick days	-0.0002	0.0001	-0.0002	0.0000	0.0367	0.0005
Bed days	0.0023*	-0.0001	0.0017*	-0.0001	0.0777	0.0003
Off-work days	0.0009*	-0.0001	0.0006*	0.0000	0.1085*	0.0006
Good health	0.0026*	-0.0005	0.0029*	-0.0004	0.3327	-0.0002
Average health	0.0205*	-0.0093	0.0192*	-0.0076	1.9427*	0.0002
Poor health	0.0513*	-0.0060	0.0482*	-0.0049	3.8084*	0.0030
Very poor health	0.0760*	-0.0007	0.0590*	-0.0005	4.5021*	0.0014
Married	0.0218*	0.0048	0.0211*	0.0041	-4.2129*	-0.0031
Divorced	0.0323*	-0.0012	0.0264*	-0.0009	-1.9405	0.0005
widowed	0.0366*	-0.0078	0.0405*	-0.0075	-2.7708*	0.0015
Elementary	0.0055*	-0.0033	0.0041*	-0.0021	-0.3005	0.0002
Primary	0.0059*	0.0053	0.0037*	0.0029	-0.6817	-0.0012
High	0.0079*	0.0063	0.0048*	0.0033	-0.6855	-0.0011
University	-0.0013	-0.0002	-0.0039	-0.0006	-0.5470	-0.0003
farmer	0.0028	-0.0051	0.0026	-0.0040	-1.4257*	0.0071
student	-0.0069*	-0.0029	-0.0054*	-0.0020	-1.2727	-0.0006
Other occupations	-0.0028	-0.0036	-0.0026	-0.0028	-0.5469	-0.0019
Central region	-0.0024*	0.0003	-0.0017	0.0002	2.5443*	-0.0003
Western region	0.0024*	-0.0050	0.0021	-0.0038	4.0404*	-0.0330
CMS/NCMS	-0.0036*	-0.0014	-0.0024*	-0.0008	0.7690	0.0017
Other medical insurance	0.0085*	0.0029	0.0085*	0.0025	1.7101*	0.0028
Time	0.0017*	-0.0047	0.0022*	-0.0052	0.8036*	-0.0062
Price of outpatient	0.0037*	0.0037	0.0038*	0.0033	-0.4106	-0.0010
Price of inpatient	-0.0091*	-0.0154	-0.0098*	-0.0144	2.3316*	0.0196
LR Chi2	4510.55		4566.26		872.55	
P	< 0.001		< 0.001		< 0.001	

Note: There are 112116 observations when decomposing the inequality of probability of inpatient visit and inpatient visits in 2003, and 3742 observations when decomposing the Per-visit hospitalization days in 2003. Marginal effects which differ significantly from zero (at p < 0.05) in the regressions are showed with *.

**Table 4:** Decomposition of inequality in the inpatient utilization in 2008

	Prob of inpatient visit		Inpatient visits		Per-visit hospitalization days	
			
	Marginal effects	Contributions	Marginal effects	Contributions	Marginal effects	Contributions
Income	0.0244*	0.1462	0.0246*	0.1213	2.7223*	0.1013
Male	-0.0125*	0.0003	-0.0095*	0.0002	3.2477*	0.0015
Age 35-44	-0.0357*	-0.0144	-0.0316*	-0.0105	2.7352*	0.0045
Age 45-54	-0.0320*	0.0025	-0.0282*	0.0018	3.0854*	0.0016
Age 55-64	-0.0261*	0.0061	-0.0218*	0.0042	2.2320*	-0.0026
Age 65+	-0.0210*	0.0067	-0.0149*	0.0039	1.2718*	-0.0014
Illness in last two weeks	-0.0024	0.0001	-0.0009	0.0000	-2.3136*	-0.0015
Chronic disease	0.0591*	-0.0001	0.0667*	-0.0001	0.5897*	-0.0001
Sick days	0.0005	-0.0002	0.0004	-0.0001	0.1635*	0.0017
Bed days	0.0036*	0.0002	0.0023*	0.0001	0.1303*	0.0014
Off-work days	0.0020*	0.0003	0.0011*	0.0001	-0.0650	-0.0003
Health status index	-0.0006*	-0.0102	-0.0007*	-0.0089	-0.0575*	0.0019
Married	0.0393*	0.0047	0.0358*	0.0035	-4.6326*	-0.0036
Divorced	0.0544*	-0.0007	0.0653*	-0.0007	-0.8367	0.0000
widowed	0.0429*	-0.0064	0.0347*	-0.0043	-3.4078*	0.0029
Elementary	0.0098*	-0.0036	0.0090*	-0.0027	-0.5663*	0.0008
Primary	0.0112*	0.0053	0.0086*	0.0033	-1.1152*	-0.0023
High	0.0108*	0.0040	0.0088*	0.0027	-0.8167*	-0.0011
University	0.0129	0.0011	0.0126	0.0009	-1.2779	-0.0005
farmer	0.0004	-0.0004	0.0032	-0.0024	-0.3973	0.0021
student	-0.0267*	-0.0052	-0.0271*	-0.0044	-1.7528*	-0.0006
Other occupations	-0.0061*	-0.0054	-0.0035	-0.0025	-0.3558	-0.0015
Central region	0.0113*	-0.0003	0.0099*	-0.0002	1.2769*	0.0005
Western region	0.0097*	-0.0127	0.0104*	-0.0112	1.7177*	-0.0121
NCMS	0.0140*	-0.0039	0.0146*	-0.0033	1.7653*	-0.0031
Other medical insurance	0.0191*	0.0046	0.0220*	0.0044	5.5013*	0.0085
Time	0.0000	0.0000	-0.0002	0.0002	0.4640*	-0.0032
Price of outpatient	0.0016	0.0011	0.0009	0.0005	-0.5445*	-0.0027
Price of inpatient	-0.0202*	-0.0255	-0.0212*	-0.0221	0.9523*	0.0086
LR Chi2	5629.35		5974.71		1640.16	
P	< 0.001		< 0.001		< 0.001	

Note: There are 103754 observations when decomposing the inequality of probability of inpatient visit and inpatient visits in 2008, and 6373 observations when decomposing the Per-visit hospitalization days in 2008. Marginal effects which differ significantly from zero (at p < 0.05) in the regressions are showed with *.

Among these contributions, income made the greatest contribution to the inequality of inpatient utilization in each year and all of the contributions were positive, indicating that most of the pro-rich inequalities are accounted for by income. The contributions of need variables on the inequality of probability of an inpatient visit and the number of inpatient visits are negative in 2003 and 2008, meaning that poorer individuals have greater need, while the contributions on the inequality of per-visit hospitalization days are positive, suggesting that the wealthy have greater need of hospitalization. For the inpatient utilization in 2003 and 2008, the contributions of other variables on inequality were negative, of which inpatient price made the most contribution to the pro-poor inequality of the probability of an inpatient visit and inpatient visits (table 2). This is perhaps because the inequality of inpatient price is very small for rich and poor. The residual term made positive contributions on the inequality of all inpatient utilization in each year except the per-visit hospitalization days in 2003.

### Decomposition of change in the concentration index

Table 1 shows that, from 2003 to 2008, the concentration indices of probability of an inpatient visit and the number of inpatient visits decreased by 48.79 and 44.49% respectively, meanwhile, the concentration index of per-visit hospitalization days increased by 16.47%. In order to find out which factors resulted in the change of inequality, the method of decomposing the change in concentration index was employed to analyze the contributions of each determinant on the change of inequality in inpatient utilization (table 5). Like each variable's contribution to inequality, the absolute value of the contribution to the change of inequality signifies to what extant the change can be attributed to each variable. The variable contributes to the increase of pro-rich inequality if the value of the contribution is positive, which means the gap of utilization between the rich and poor will increase, and vice versa.

**Table 5:** Contributions of the change of inequality in inpatient utilization from the 2003 to 2008

	Prob of inpatient visit	Inpatient visits	Per-visit hospitalization days
Socioeconomic status	-0.0828	-0.0952	-0.0122
Male	0.0054	0.0045	-0.0058
Age 35-44	0.0042	0.0038	-0.0009
Age 45-54	0.0060	0.0048	0.0020
Age 55-64	0.0073	0.0052	-0.0010
Age 65+	0.0052	0.0021	-0.0003
Illness in last two weeks	0.0012	0.0006	0.0018
Chronic disease	-0.0150	-0.0167	0.0024
Sick days	-0.0028	-0.0022	-0.0053
Bed days	-0.0002	-0.0001	0.0010
Off-work days	0.0004	0.0002	-0.0002
Married	-0.0229	-0.0183	0.0026
Divorced	-0.0003	-0.0008	-0.0004
widowed	-0.0040	-0.0002	0.0031
Elementary	-0.0046	-0.0045	0.0018
Primary	-0.0019	-0.0018	0.0008
High	-0.0006	-0.0003	0.0001
University	0.0005	0.0005	0.0000
farmer	0.0051	-0.0006	-0.0134
student	0.0004	0.0006	0.0002
Other occupations	-0.0031	-0.0010	0.0003
Central region	-0.0070	-0.0056	0.0036
Western region	-0.0167	-0.0163	0.0302
CMS/NCMS	-0.0586	-0.0560	-0.0456
Other medical insurance	0.0028	0.0027	0.0039
Time	0.0095	0.0123	0.0118
Price of outpatient	0.0130	0.0183	0.0076
Price of inpatient	0.1431	0.1410	0.0993

**Figure 3:**
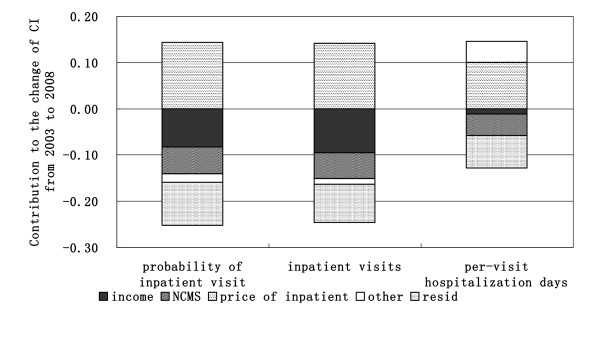
**Decomposition of the change of inequality in inpatient utilization from 2003 to 2008**.

From Figure 3, we found that, the variables of income, NCMS and inpatient price made great contributions to the change of inequality in inpatient utilization, from which income and NCMS contributed to the increase of pro-poor inequality and inpatient price contributed to the increase of pro-rich inequality. In addition, residual variables made big contributions to the increase of pro-poor inequality and other variables made little contributions to the change of inequality.

## Discussion

Using the data from Chinese National Health Services Surveys (NHSS), this paper provided the first analysis of income-related inequality and inequity of inpatient utilization in rural China. The horizontal inequity index of inpatient service utilization in 2003 and 2008 shows that obvious pro-rich inequities of inpatient utilization exist in rural China, which indicates that a disproportionate share of inpatient resources are utilized by richer people in spite of lower need. These results are consistent with previous studies [20,21]. The results suggest, for instance, that the inequity of the number of inpatient visits is a little bit larger than the inequity of the probability of an initial inpatient visit. The inequity of the number of inpatient visits in rural China is therefore mainly generated by the initial inpatient visit rather than subsequent visits. Poorer individuals are far less likely to make an initial inpatient visit. However, those that make an initial visit are only slightly less likely than richer individuals to make subsequent visits. From 2003 to 2008, as the factors associated with inpatient utilization changed (e.g., income, inpatient price and coverage rate of medical insurance), the inequities of the probability of inpatient visit and inpatient visits decreased 48.27% and 43.59% respectively. This suggests that in spite of growing costs, improving income and greater health insurance coverage offset the increased price and have reduced income-related inequities in inpatient utilization. On the other hand, the inequity of per-visit hospitalization days increased by 29.96%. There are two probable reasons for this increase in inequity. First, as the payment method is fee-for-service in hospitals, with the increase of price, poor individuals are more likely to shorten their number of hospitalization days compared with the rich even though the inpatient expenditure can be partly paid by health insurance. Second, although more than 90% of rural residents were enrolled in NCMS in 2008, the reimbursement rates of NCMS in primary hospitals are much higher than in high-level hospitals, but there are more average hospitalization days in high-level hospitals than in primary hospitals in China. As a result, poor enrollees are more likely to be hospitalized in primary hospitals in 2008 than in 2003 where they can get a higher reimbursement rate and richer individuals are more likely to be hospitalized in high-level hospitals where they will likely spend more days in the hospital. Consequently, poorer individuals will spend fewer days in the hospital than richer individuals even if their number of visits is the same.

After decomposing the inequality of inpatient utilization, we find that income made the greatest pro-rich contribution to the inequality of inpatient utilization. In other words, rising incomes between 2003 and 2008 increased income differentials in utilization rates. As previously noted, the contribution of income is equal to the product of the elasticity of inpatient utilization and the inequality of residents' income (measured as the income Gini coefficient). Because we cannot change the elasticity of inpatient price, the only effective way to increase the equity of inpatient utilization is to reduce the inequality of residents' income. As table 6 demonstrates, the income Gini coefficient was larger in 2003 than in 2008 (0.369 and 0.363 respectively), which is consistent with the fact that the inequities in the probability of an inpatient visit and number of inpatient visits in 2003 is greater than 2008. The contributions of need variables to the inequality of inpatient utilization are similar in 2003 and 2008. In both years, the contributions to the probability of an inpatient visit and number of inpatient visits are in a pro-poor direction and the contribution to per-visit hospitalization days is in a pro-rich direction. This indicates that the poor need relatively more inpatient utilization, but that rich individuals likely need more per-visit hospitalization days than the poor. A potential explanation for this finding is that poor rural individuals are more likely to suffer from diseases that require more inpatient visits than rich individuals, but rich individuals suffer more from chronic disease that requires longer term care than poor individuals (i.e., rich individuals are more likely to develop chronic diseases, such as hypertension, diabetes and coronary heart disease, than poor individuals in rural China). Thus, need has stayed relatively constant even as inequity has reduced.

**Table 6:** Description of main determinants and their concentration indexes in 2003 and 2008

	2003		2008	
		
	Means	CI	Means	CI
Income (RMB)	2690	0.369*	4471	0.363*
CMS/NCMS (%)	9.67	-0.016	90.24	-0.019
Inpatient price (RMB)	1473	0.008	1977	0.010

Note: * Gini coefficient is based on income.

Theoretically, the contribution of each determinant to the change of concentration index of inpatient utilization can be attributed to an interaction of changes, which includes the change of this determinant, the change of the determinant' concentration index, and the change of partial effects of the determinant on inpatient utilization. The results show that the main determinants of the change are improving income, NCMS and inpatient price. Therefore, the increase in income, the decrease in pro-rich inequality of income, the improvement of the coverage rate of NCMS and the decrease in pro-rich inequality of NCMS made a great contribution to reduce the pro-rich inequality from 2003 to 2008. Meanwhile, the increase in inpatient price and the pro-rich inequality of inpatient price made a great contribution to increase the pro-rich inequality (see table 6). Furthermore, the results also show that residual variables contributed a lot to the increase in pro-poor inequality, suggesting that there remains a good deal of unexplained variation in changes in inequity beyond the variables examined in this analysis.

There are several limitations to this study which should be noted. Firstly, health status, which is used to measure health care need, is self-reported. Self-reported health status can be considerably affected by residents' health consciousness level and health knowledge level. For instance, poor people's self-reported health status may better than their actual health status because they lack health consciousness and knowledge to make accurate assessments compared with wealthier individuals. Therefore, the equity of inpatient utilization may be underestimated in this paper. Secondly, due to the impossibility of attaining actual medical prices in each region, we assumed that the medical prices are identical in the county level, and measured outpatient price and inpatient price by the medians of per-visit outpatient expenses and inpatient expenses in each county. The rationale for this assumption should be further validated.

## Conclusion

There is a strong pro-rich inequity of inpatient utilization in Chinese rural areas. The horizontal equity of both the probability of initial inpatient visit and total number of visits greatly improved from 2003 to 2008, compared with a decline in the horizontal equity of per-visit hospitalization days. As income was the main factor contributing to the pro-rich inequality of inpatient utilization, this study suggests that an effective way to reduce the inequity is to narrow the gap of income between the rich and poor. As income, NCMS and the inpatient price were highly associated with the change of inequality in inpatient utilization from 2003 to 2008, improved income, a narrowed gap of income between the rich and the poor and an increased coverage rate of NCMS (especially for poor individuals) have effectively facilitated the improvement of equity in inpatient utilization. Meanwhile, the increased inpatient price and its increased inequality in rural China have hindered the improvement of the equity in inpatient utilization.

## Competing interests

The authors declare that they have no competing interests.

## Authors' contributions

ZZ participated in the design, data analysis and was the primary person responsible for drafting the manuscript. JG participated in the design, data analyses and review. AF participated in writing, review and revision. KR participated in the design, data collection and data analysis. KX participated in the design and review. LX and YZ participated in the data collection and review. All authors read and approved the final manuscript.

## Pre-publication history

The pre-publication history for this paper can be accessed here:

http://www.biomedcentral.com/1472-6963/11/201/prepub
